# Supplementary material for: Clinical Utility of 4C Mortality Scores among Japanese COVID-19 Patients: A Multicenter Study
Source: J Clin Med. 2022 Feb 3;11(3):821. doi: 10.3390/jcm11030821 (PMC8836893; doi:10.3390/jcm11030821)
Supplement: Supplementary file 1 [file jcm-11-00821-s001.zip › jcm-1554978-supplementary.pdf]

Table S1. Comparison of use of respiratory support in the COVID-19 patients, by outcomes.

	**Overall**	**Survived**	**Dead**	***p*-value**
Use of respiratory support (%)				
High flow nasal cannula	33 (16)	28 (17.8)	5 (23.8)	0.34
Non-invasive MV	1 (0.5)	1 (4.8)	0 (0)	0.10
Invasive MV	37 (18.0)	28 (15.1)	9 (42.5)	0.004
ECMO	3 (1.46)	1 (0.76)	2 (9.5)	0.03

Shown are the respiratory supports that the patients were provided at their worst conditions. MV, mechanical ventilation; ECMO, extracorporeal membrane oxygenation.
